# Intraoperative Blood Flow Analysis of Free Flaps with Arteriovenous Loops for Autologous Microsurgical Reconstruction

**DOI:** 10.3390/jcm12237477

**Published:** 2023-12-02

**Authors:** Alexander Geierlehner, Raymund E. Horch, Ingo Ludolph, Werner Lang, Ulrich Rother, Alexander Meyer, Andreas Arkudas

**Affiliations:** 1 Department of Plastic and Hand Surgery and Laboratory for Tissue Engineering and Regenerative Medicine, University Hospital Erlangen, Friedrich Alexander University Erlangen-Nürnberg FAU, 91054 Erlangen, Germany; raymund.horch@uk-erlangen.de (R.E.H.); ingo.ludolph@fau.de (I.L.); andreas.arkudas@uk-erlangen.de (A.A.); 2 Department of Vascular Surgery, University Hospital Erlangen, Friedrich Alexander University Erlangen-Nürnberg FAU, 91054 Erlangen, Germany; werner.lang@uk-erlangen.de (W.L.); ulrich.rother@uk-erlangen.der (U.R.); alexander.meyer3@helios-gesundheit.de (A.M.); 3 Department of Vascular Surgery and Phlebology, Helios Klinikum Berlin-Buch, 13125 Berlin, Germany

**Keywords:** reconstructive surgery, plastic surgery, microsurgery, surgical innovation, ICG fluoroscopy, interdisciplinary plastic surgery

## Abstract

Background: Arteriovenous (AV) loops help to overcome absent or poor-quality recipient vessels in highly complex microvascular free flap reconstruction cases. There are no studies on blood flow and perfusion patterns. The purpose of this study was to evaluate and compare intraoperative hemodynamic characteristics of AV loops followed by free tissue transfer for thoracic wall and lower extremity reconstruction. Methods: this prospective clinical study combined Transit-Time Flowmetry and microvascular Indocyanine Green Angiography for the assessment of blood flow volume, arterial vascular resistance and intrinsic transit time at the time of AV loop construction and on the day of free flap transfer. Results: A total of 11 patients underwent AV loop creation, of whom five required chest wall reconstruction and six required reconstruction of the lower extremities. In seven of these cases, the latissimus dorsi flap and in four cases the vertical rectus abdominis myocutaneous (VRAM) flap was used as a free flap. At the time of loop construction, the blood flow volume of AV loops was 466 ± 180 mL/min, which increased to 698 ± 464 mL/min on the day of free tissue transfer (*p* > 0.1). After free flap anastomosis, the blood flow volume significantly decreased to 18.5 ± 8.3 mL/min (*p* < 0.001). There was no significant difference in blood flow volume or arterial vascular resistance between latissimus dorsi and VRAM flaps, nor between thoracic wall and lower extremity reconstruction. However, a significant correlation between the flap weight and the blood flow volume, as well as to the arterial vascular resistance, was found (*p* < 0.05). Conclusion: This is the first study to perform intraoperative blood flow and hemodynamic measurements of AV loops followed by free tissue transfer. Our results show hemodynamic differences and contribute to deeper understanding of the properties of AV loops for free flap reconstruction.

## 1. Introduction

Split-thickness skin graft (STGT), full-thickness skin graft (FTGT), as well as local and regional flaps, very often count as the most suitable procedure for reconstructive purposes [1,2]. However, such procedures are usually unsuccessful for large, complex defects, making free flap transfer inevitable. Microsurgical free tissue transfer is defined as the highest order in the reconstructive ladder, and is considered a highly sophisticated and demanding procedure [3]. Despite its technical challenges, free flaps have proven to be a safe and reliable method. The literature indicates that the causes of free flap failure and revision rates are multifactorial. This includes flap-related and patient-related causes such as a poor vascular status [4,5,6,7]. Sufficient donor vessels close to the area of the defect is a basic prerequisite for the success of free flaps. Unfortunately, recipient vessels can be absent or inadequate for different reasons, including tissue irradiation, significant trauma or peripheral arterial disease. The arteriovenous (AV) loop, first described in 1982, overcomes this limitation by creating a neovessel using a vein graft anastomosed to a sufficient arterial and venous vessel close to the defect outside the zone of injury [8,9,10]. The AV loop provides an adequate caliber and serves as a newly created recipient vessel for free flap transfer. AV loop creation and free flap transfer can either be performed as a single surgical procedure (single-staged approach) or as two separate surgical interventions with the free tissue transfer usually performed between one and two weeks after AV loop placement (two-staged approach) [11]. The efficacy of free flap reconstruction combined with an AV loop in terms of patient outcome, limb salvage and flap survival has been confirmed in the literature by several study groups [12,13,14,15,16]. Despite its successful application, it must be noted that this type of free flap surgery is reserved for a highly specialized patient population, with thromboembolic events still remaining a common risk factor for flap failure. Numerous technological tools have been developed to allow visualization and measurement of vascularity and perfusion of transferred tissue at a pre-, intra- and postoperative level to enhance its safety and efficacy [17,18,19,20,21,22,23]. However, to date, there is no data on intraoperative perfusion characteristics of free flaps with AV loops. Our objective was to perform intraoperative blood flow measurements in patients receiving an AV loop followed by free tissue transfer. To the best of our knowledge, this is the first study to obtain crucial intraoperative blood flow parameters such as blood flow volume, arterial vascular resistance and intrinsic transit time of AV loops followed by free tissue transfer. In doing so, it allows comparative evaluation of different free flap types with AV loops for thoracic wall and lower extremity reconstruction. For this purpose, we used a previously published method by our study group combining Transit-Time Flowmetry (TTFM) and microvascular Indocyanine Green Angiography (mICG-A) [24]. This study aims to establish normative blood flow and flap perfusion values serving as groundwork for the determination of predictive values for postoperative thrombotic events in this highly selective patient population.

## 2. Materials and Methods

This prospective mono-centered cohort study included patients receiving free tissue transfer in combination with arteriovenous (AV) loops for autologous microsurgical reconstruction from March 2020 to March 2023. Patients with a lack of sufficient donor vessels hence requiring an arteriovenous loop as last resort for free flap reconstruction of the thoracic wall or lower extremities were eligible for study inclusion. Patients in whom an AV Loop creation could not be performed for technical reasons were excluded from this study. In addition, patients where the AV loop could not be used due to recurrent thromboses prior to free flap reconstruction were excluded. The study was approved by the Ethical Committee of the Friedrich-Alexander-University Erlangen-Nürnberg (Registration Number: 447_19B) in accordance with the Declaration of Helsinki. The study was conducted according to the STROBE guidelines (Supplementary Table S1). Written consent was given by each patient prior to study inclusion.

### 2.1. Surgical Technique

All patients suffering from sternal defects received a standardized computed tomography angiography (CTA) of the neck and the thoracic wall. Patients requiring lower extremity reconstruction received a digital subtraction angiography (DSA) prior to surgical intervention. Only cases with missing or inadequate recipient vessels were chosen to be eligible for AV loop construction and for study inclusion. All included patients received surgical wound debridements combined with negative pressure wound therapy (NPWT) for at least one week prior to free tissue transfer. In all cases, microsurgical reconstruction was performed in a two-staged procedure. In principle, it is possible to perform both steps (AV loop creation and free flap transfer) in a single surgical procedure. However, in such critically ill patients with long-standing defects and prolonged bed rest, experience has shown that unexpected venous thrombosis proximal to the AV loop, which is not directly clinically apparent, may occur. Thus, a two-stage procedure allows surgical intervention of AV loop thrombosis before free tissue transfer has even been performed. This reduces the risk of free flap loss. Therefore, no patients in this study underwent a single-stage procedure, as this is no longer carried out at our clinic. First, experienced vascular surgeons at the University Hospital Erlangen performed the AV loop construction close to the area of the defect using an autologous great saphenous vein graft in all cases. Prior to free flap reconstruction, AV loop patency was confirmed by color-coded duplex sonography, CTA or DSA. Afterwards, six to nine days after AV loop construction, the microsurgical free flap reconstruction using the AV loop as recipient vessel was performed by three experienced plastic surgeons at our Department at the University Hospital Erlangen. The included patients received either a latissimus dorsi or a vertical rectus abdominis myocutaneous (VRAM) free flap. Flap harvest was performed in a standardized fashion. The AV loop was dissected at the apex. The arterial and venous AV loop parts were then anastomosed in an end-to-end fashion with the artery and vein of the flap pedicle, respectively. Arterial anastomoses were all hand-sewn. Whereas venous anastomoses were either hand-sewn or coupled using a venous coupler device (Synovis Micro Companies Alliance, Inc., Birmingham, AL, USA). Patients with a known allergy/hypersensitivity to Indocyanine green or sodium iodide were excluded from this study. Also, patients suffering from hyperthyroidism, thyroid adenoma or autonomy were not eligible for study inclusion. During free flap reconstruction, the patient’s body temperature was kept stable by maintaining the OR temperature between 20 °C and 22 °C and by using a warming mattress of 37 °C. The included patients received either unfractionated heparin as a perfusor or low molecular weight heparin postoperatively.

### 2.2. Transit-Time Flowmetry (TTFM)

Transit-Time Flowmetry, an ultrasound-based technology, enables the measurement of blood flow volume within a vessel. Previous studies have shown a high correlation (r = 0.93–0.95) with the actual blood flow volume, making it a reliable tool for measurements at a microsurgical level [24,25]. In this study, measurements were performed with MiraQTM Vascular (Medistim ASA, Solo, Norway) at predefined intraoperative time points (Table 1).

### 2.3. Arterial Vascular Resistance (aVR)

The calculation of the arterial Vascular Resistance as millimeters of mercury per milliliter per minute (mmHg/mL/min) is based on a commonly reported method in accordance with the Poiseuille’s Law, resulting in the following formula [24,26,27]

aVR=MAP/aBF

*aVR = arterial Vascular Resistance* (mmHg/mL/min)

*MAP = Mean Arterial Pressure* (mmHg)

*aBF = arterial Blood Flow* (mL/min)

The Mean Arterial Pressure (MAP; in mmHg) is the mean arterial blood pressure of a single cardiac cycle which was measured and documented at each measurement time point (Table 1).

### 2.4. Microvascular Indocyanine Green Angiography (mICG-A)

**Figure 1:**
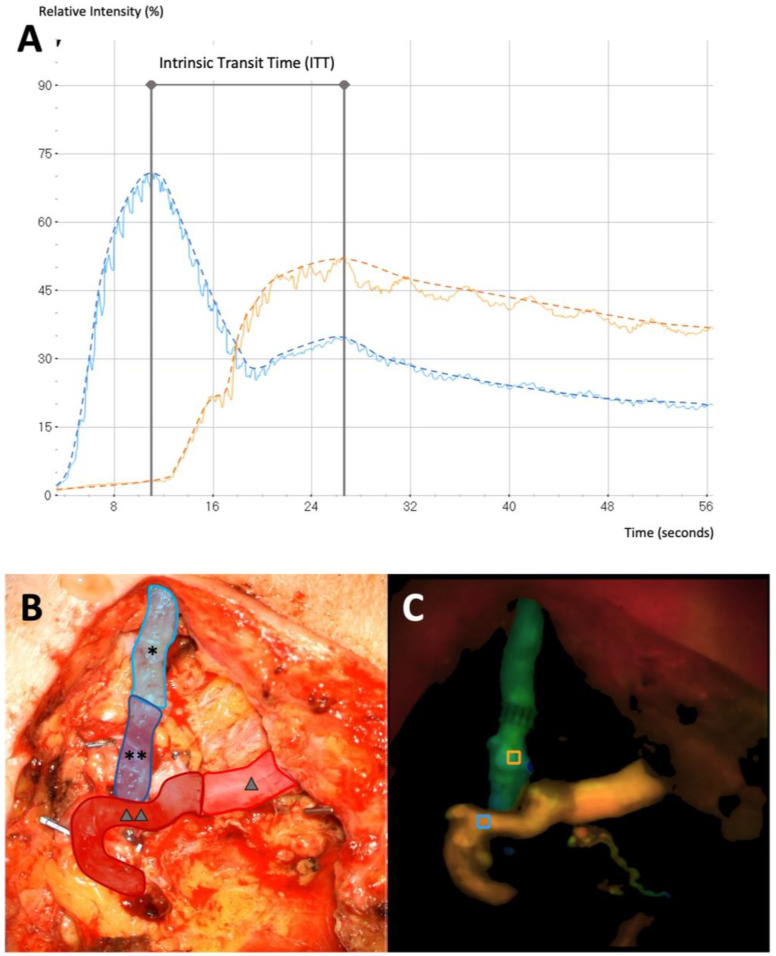
(**A**) Microvascular Indocyanine Green Angiography (mICG-A) flow curves in two selected regions of interest (ROI) (blue curve: arterial flow, orange curve: venous flow). The time between maximum fluorescence intensity of the arterial and the venous flow delineates the ITT. (**B**) Intraoperative image after anastomosis of the AV loop with the free flap (light blue (*): venous segment of the AV loop, dark blue (**): free flap vein, light red (∆): arterial segment of the AV loop, dark red (∆∆): free flap artery. (**C**) Delay Map obtained with FLOW800 illustrating both ROIs (blue ROI placed at the artery, orange ROI placed at the vein) and picturing the two flow curves.

After arterial and venous anastomosis of the flap pedicle with the AV loop, the fluorescent dye Indocyanine Green (VERDYE, Diagnostic Green, Munich, Germany) was administered as an intravenous bolus of 3 mL (2.5 mg/mL) (Table 1). The microscope integrated software FLOW800 (KINEVO 900 Version 1.8, Carl Zeiss, Oberkochen, Germany) allows real-time blood flow imaging by visualization of the fluorescent dye within blood vessels [28,29,30]. Color-coded delay maps then display the temporal latency of the maximum fluorescence intensity. The software calculates the maximum fluorescence intensity at manually placed regions of interest (ROI). The flap artery and vein close to the anastomosis uncovered from any surrounding soft tissue were chosen as ROIs in this study. The time between the maximum ICG intensity at the arterial inflow and the venous outflow of the flap is defined as Intrinsic Transit Time (ITT) (Figure 1). It is considered as the passage time required for the contrast medium to traverse the vascular network of the transferred tissue and is thus a measure of blood flow velocity within the flap.

### 2.5. Postoperative Hemodynamic Complications

The patient follow-up was at least 3 months. Hemodynamic complications included early and late venous and arterial thrombosis, as well as flap failure due to a thromboembolic event. Cases of early arterial or venous thrombosis required immediate surgical revision. A late thrombosis was defined as a thromboembolic event with a clinically recognizable impaired perfusion of the transferred tissue after hospital discharge. Cases of late thrombosis required color-coded duplex sonography, CTA or DSA to confirm vascular compromise of the AV loop or the flap pedicle. In such cases, Indocyanine Green Angiography was additionally performed to evaluate the extent of perfusion impairment of the free flap.

### 2.6. Statistical Analysis

Descriptive analysis was performed for patient demographics and postoperative hemodynamic complications. Values are presented as mean ± standard deviation. Parametric data within one group were compared using the paired Student’s *t*-test. Parametric data of two different groups were analyzed using an unpaired Student’s *t*-test. Nonparametric data were analyzed with the Wilcoxon matched-pairs rank test within one group, whereas the Mann–Whitney U test was used for analyses of nonparametric data between two different groups. The correlation of data assuming Gaussian distribution was calculated using the Pearson correlation coefficient. The Spearman’s rank correlation coefficient was used for data not passing a test for normality. The significance level (*) was set at *p* ≤ 0.05, whereas *p*-values ≤ 0.001 were considered highly significant (**). Outliers were identified using the ROUT method (Q = 10%) and appropriately excluded from statistical analysis. Statistical analyses and graphic illustrations were performed using GraphPad Prism (GraphPad Software Version 6.0, Inc., San Diego, CA, USA).

## 3. Results

**Figure 2:**
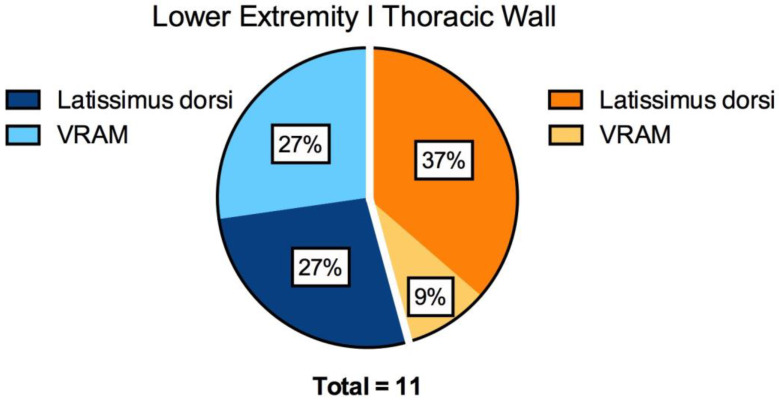
Pie chart illustrating the percentage of latissimus dorsi and VRAM flaps for lower extremity and thoracic wall reconstruction.

A total of 11 patients (five female, six male) receiving an AV loop followed by free flap reconstruction of the thoracic wall or the lower extremity were included in this prospective study. The AV loop implementation and the free tissue transfer were performed in two separate procedures as a two-staged approach. The average time interval between these two surgeries was 7 days, ranging from 6 to 9 days. The average patient age was 63.6 years, ranging from 27 to 80 years. Five patients required the reconstruction of the thoracic wall (45%) and six patients lower extremity reconstruction (55%) (Figure 2). Of the included patients, seven received a latissimus dorsi muscle (64%) and four received a VRAM (36%) free flap. In total, four latissimus dorsi flaps (37%) and one VRAM flap (9%) were used for the reconstruction of the thoracic wall. In contrast, three patients received a latissimus dorsi (27%) and three patients a VRAM flap (27%) for lower extremity reconstruction (Figure 2). In all cases, the great saphenous vein was used as AV loop graft. In all six lower extremity reconstruction cases, the AV loop was anastomosed with the femoral vessels. In all five cases of thoracic wall reconstruction, the loop was anastomosed with the subclavian vessels. All arterial anastomoses and 6 out of 11 venous anastomoses were hand-sewn. A total of five venous anastomoses were performed using a microvascular anastomotic coupler system with a 3.5 or 4 mm diameter coupler ring. The average flap weight was 525 ± 222 g. The average weight of latissimus dorsi flaps was 441 ± 193 g whereas the average VRAM flap weight was 651 ± 224 g. The average flap ischemia time was 61 ± 12 min (Supplementary Table S2).

### 3.1. Blood Flow Volume (mL/min)

**Figure 3:**
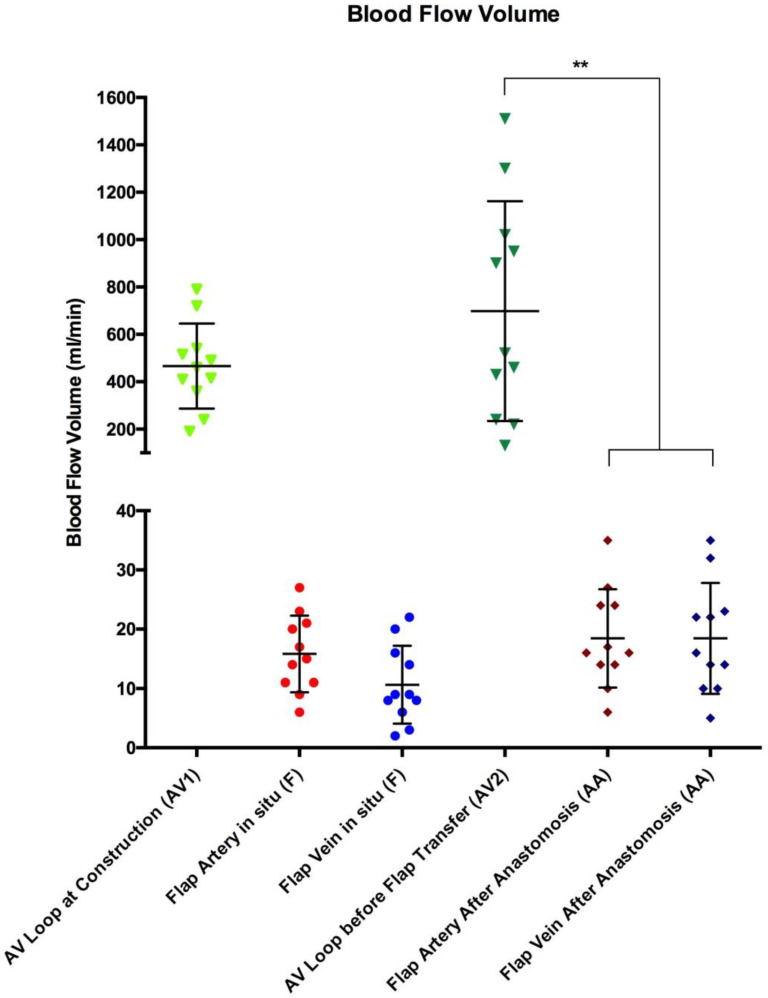
Blood flow volume (mL/min) of AV loops and free flaps at predefined time points (AV1, F, AV2 and AA). Error bars represent means ± standard error (** indicates highly significant differences). AV1 (light green), Flap Artery in situ (light red), Flap Vein in situ (light blue), AV2 (dark green), Flap Artery After Anastomosis (dark red), Flap Vein After Anastomosis (dark blue).

**Figure 4:**
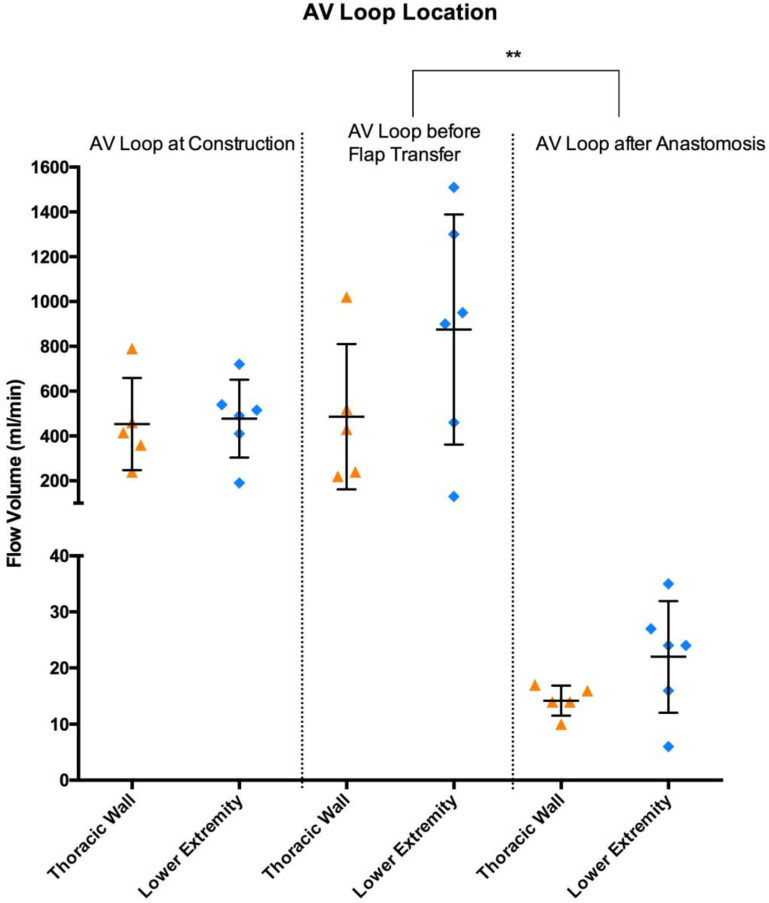
Blood flow volume (mL/min) of AV loops at both regions of reconstruction (thoracic wall (orange) and lower extremity (light blue)) at three different time points (at construction (AV1), before flap transfer (AV2) and after flap anastomosis (AA)). Error bars represent means ± standard error (** indicates highly significant differences).

At the time of AV loop construction (first surgical procedure), the intraoperative blood flow volume of AV loops was 466 ± 180 mL/min. On the day of free tissue transfer (second surgical procedure), blood flow increased to a volume of 698 ± 464 mL/min (*p* > 0.1). The average arterial blood flow volume of all flaps prior to tissue transfer was 15.8 ± 6.4 mL, with an average venous flow volume of 10.6 ± 6.6 mL/min (Table 2). The blood flow volume of the included flaps did not significantly change after free tissue transfer and anastomosis with the AV loop (arterial flow volume of 18.5 ± 8.3 mL/min and venous flow volume of 18.5 ± 9.3 mL/min) (Figure 3). However, blood flow volume of all AV loops, irrespective of its location, decreased highly significantly from 698 ± 464 mL/min to 18.5 ± 8.3 mL/min after free flap anastomosis (*p* < 0.001) (Figure 3 & Table 3). The blood flow volume of AV loops for thoracic wall reconstruction increased from 453 ± 206 mL/min at the time of loop creation to 486 ± 324 mL/min at the day of reconstruction prior to free flap anastomosis. AV loops for lower extremity reconstruction showed an increase in blood flow volume from 478 ± 174 mL/min at loop construction to 875 ± 513 mL/min before free flap transfer (Figure 4). There was no significant difference in blood flow volume between the AV loops at construction for thoracic wall and lower extremity reconstruction. The blood flow volume of the femoral AV loop for lower extremity reconstruction was higher, yet not significantly higher, than the subclavian AV loop for thoracic wall reconstruction both before and after free flap anastomosis (Figure 4). The blood flow volume of both the femoral and the subclavian AV loop decreased highly significantly after free tissue transfer (*p* < 0.001) (Figure 4). The arterial blood flow volume of latissimus dorsi flaps in situ (16.6 ± 6.9 mL/min) did not change significantly after anastomosis with the high-flow AV loop system (780 ± 495 mL/min) remaining at an average of 16.3 ± 7.4 mL/min. VRAM flaps showed similar results with an initial blood flow volume of 14.5 ± 6.2 mL/min in situ. After anastomosis with the AV loop (555 ± 430 mL/min) the arterial blood flow remained at an average volume of 22.3 ± 9.5 mL/min. There was no significant difference in terms of blood flow volume between latissimus dorsi and VRAM flaps neither before nor after anastomosis.

### 3.2. Arterial Vascular Resistance (mmHg/mL/min)

**Figure 5:**
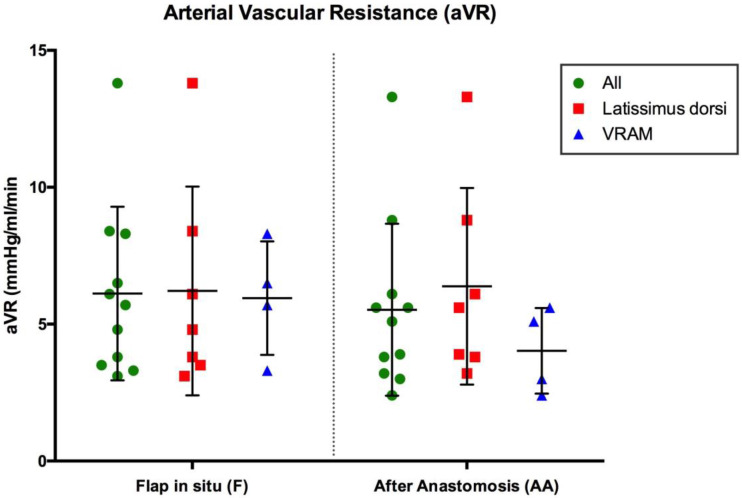
Arterial Vascular Resistance (aVR) of the flap in situ prior to tissue transfer (F) and after anastomosis (AA). Error bars represent means ± standard error.

**Figure 6:**
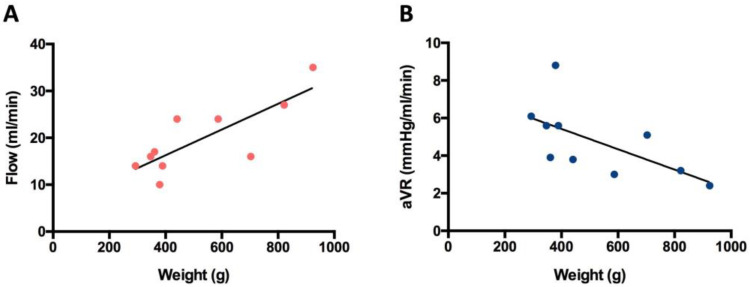
(**A**) Arterial blood flow (mL/min; red dots) after anastomosis (AA) versus flap weight (gram); y = 0.02744x + 5.304; *p* = 0.005; r2 = 0.6427. (**B**) Arterial vascular resistance (mmHg/mL/min; blue dots) after anastomosis (AA) versus flap weight (gram); y = −0.00542x + 7.593; *p* < 0.05; r2 = 0.4008.

The average arterial vascular resistance (aVR) of all included free flaps prior to tissue transfer (6.1 ± 3.2 mmHg/mL/min) did not change significantly after AV loop anastomosis (5.5 ± 3.1 mmHg/mL/min). Latissimus dorsi flaps had an average arterial vascular resistance of 6.2 ± 3.8 mmHg/mL/min before and 6.4 ± 3.6 mmHg/mL/min after AV loop anastomosis. The aVR of VRAM flaps was 6 ± 2.1 mmHg/mL/min before and 4 ± 1.6 mmHg/mL/min after AV loop anastomosis and thus slightly lower than the aVR of latissimus dorsi flaps (Figure 5). There was a significant positive correlation between flap weight and arterial blood flow volume and a significant negative correlation between aVR and flap weight after anastomosis (*p* < 0.05) (Figure 6A,B).

### 3.3. Intrinsic Transit Time

**Figure 7:**
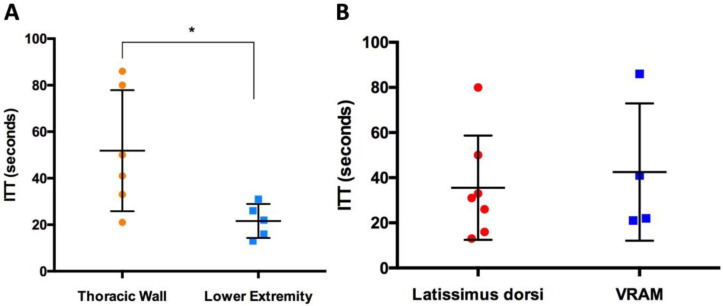
(**A**) Intrinsic Transit Time (in seconds) of free flaps after AV loop anastomosis (AA) for thoracic wall (orange) and lower extremity reconstruction (light blue). (**B**) Intrinsic Transit Time (in seconds) of latissimus dorsi flaps (red) and VRAM flaps (blue) after AV loop anastomosis (AA). (* indicates significant differences).

The average Intrinsic Transit Time (ITT) after free flap anastomosis with the AV loop was 38 ± 25 s (s). The mean ITT of latissimus dorsi flaps was 36 ± 23 s, whereas VRAM flaps showed an average ITT of 43 ± 30 s (Figure 7B). In terms of ITT, latissimus dorsi flaps did not differ significantly from VRAM flaps after AV loop anastomosis (*p* = 0.78). However, free flaps for thoracic wall reconstruction had a significantly lower ITT (22 ± 7 s) than free flaps for lower extremity reconstruction (52 ± 26 s) after anastomosis with the AV loop (*p* < 0.05) (Figure 7A). There was no correlation between the ITT and flap weight, ischemia time, arterial flow volume or the aVR after anastomosis.

### 3.4. Postoperative Hemodynamic Complications

Of all included free flaps, one latissimus dorsi free flap required surgical revision due to an early venous thrombosis on the first postoperative day. The same latissimus dorsi flap and one VRAM flap both suffered from late thrombosis at the arterial part of the AV loop 1 and 3 months postoperatively, respectively. Both patients represented with a reduced, however not completely absent, flap tissue perfusion. Thrombosis at the arterial part of the former AV loop was confirmed through DSA in both cases of lower extremity reconstruction. The overall flap survival rate was 100%. All these hemodynamic complications occurred in cases with lower extremity reconstruction. The mean arterial (11 ± 7.1 mL/min) and venous blood flow (9.5 ± 6.4 mL/min) of free flaps with late thrombosis was lower than that of free flaps without hemodynamic complications (arterial flow volume of 20.1 ± 7.9 mL/min and venous flow volume of 20.4 ± 8.9 mL/min). In contrast, the average blood flow volume of AV loops prior to free flap anastomosis was higher in cases with late venous thrombosis (1100 ± 283 mL/min) than in cases without complications (603 ± 453 mL/min). The average ITT of flaps with late venous thrombosis (46 ± 6 s) was higher, yet not significantly higher than that of flaps without hemodynamic complications (36 ± 27 s).

## 4. Discussion

The variety of reconstructive options available in plastic surgery today enables the coverage of a wide range of defects. Adequate vascular status is a basic prerequisite for local and regional as well as free flaps [1,31]. The creation of a neovessel in the form of an arteriovenous loop helps to overcome the limitation of absent, poor-quality or inadequate-caliber recipient vessels in cases requiring microvascular free flap reconstruction [12,14]. It is of utmost importance to recall that this particular procedure is reserved for a highly selective group of patients, and is usually last resort for wound closure. Hemodynamic complications remain one of the most common risk factors ultimately jeopardizing its postoperative success [12,31,32]. To date, there is no literature on the intraoperative assessment of blood flow in free flaps combined with AV loops. The current study aimed to measure and evaluate the intraoperative blood flow behavior and perfusion characteristics of AV loops with free tissue transfer. For this purpose, we combined Transit-Time Flowmetry (TTFM) with microvascular Indocyanine Green Angiography (mICG-A). The combination of these two technologies has already proven to be a reliable approach for both animal flap models and intraoperative application in humans [24,33]. Our results showed that AV loops have a blood flow volume of up to 1500 mL/min and are thus considered a high-flow system. Moreover, in the 6 to 9 days between AV loop creation and the free flap procedure (two-staged approach), the flow volume of AV loops further increased. Our study corroborates, based on our flow volume measurements, the fact that this surgical approach merges a low-flow free flap system with a high-flow AV loop system. However, after anastomosis the high blood flow of AV loops significantly decreased towards the initial blood flow volume of included flaps. Previous studies already showed that the blood flow volume of the recipient artery does not seem to have a relevant influence on the arterial blood flow of flaps after free tissue transfer. The recipient artery appears to be down- or upregulated after flap anastomosis, approximating the blood flow values of the isolated flap at its pedicle prior to tissue transfer. Since this phenomenon has been observed in fasciocutaneous, musculocutaneous and muscle flaps, it appears to be irrespective of flap tissue composition [24,34,35,36,37]. However, those previously published studies only performed free flap reconstruction using recipient vessels with low-flow characteristics. This is the first study to show that even high-flow AV loops with flow volumes of up to 1.5 L/min are downregulated towards initial blood flow volumes of the included flaps. The arterial and venous blood flow properties of both latissimus dorsi and VRAM flaps did not significantly change after anastomosis with the high-flow AV loop system. The included latissimus dorsi flaps had blood flow values similar to previously published free latissimus dorsi flaps anastomosed with the femoral, popliteal or tibial artery [35]. The arterial vascular resistance of free flaps also did not significantly change after flap revascularization and did not significantly differ between latissimus dorsi and VRAM flaps. Our results also showed that latissimus dorsi and VRAM flaps have a similar ITT. Comparison with the literature further showed that the average ITT and the arterial vascular resistance of latissimus dorsi and VRAM flaps combined in our study, both classified as myocutaneous flaps, was lower than that of fasciocutaneous flaps [24]. Our results demonstrated that the recipient vessel did not alter or even modify the perfusion properties of free flaps even at extremely high flow rates, as seen in AV loops. All these findings rather support the notion that blood flow and perfusion characteristics highly depend on the flap tissue composition and hence is a matter of vascularity of each flap type [38,39]. All the included flaps in our study were myocutaneous flaps composed mainly of muscle tissue with a known rich vascular network with large vessels. The literature asserts that the defect localization of cases with AV loops and free flap reconstruction has a decisive influence on the vessel patency and on postoperative hemodynamic complications [12,14]. In this study, all three venous thrombosis events occurred in cases with lower extremity reconstruction. The ITT, a parameter of blood flow velocity, is intended to provide an assessment of the postoperative risk of stasis-induced thrombosis after tissue transfer [40,41]. A study group by Holm et al. reported that a prolonged ITT of more than 50 s poses an increased risk for vascular compromise and surgical revision [42]. In a recently published study, we found significantly different average ITT values of DIEP (52 ± 18 s) and msTRAM flaps (33 ± 11 s). However, only one DIEP flap with the highest ITT of 77 s required surgical revision due to hemodynamic complications [24]. In this study, the ITT of latissimus dorsi flaps after AV loop anastomosis (36 ± 23 s) did not significantly differ from that of VRAM flaps after AV loop anastomosis (43 ± 30 s). However, the ITT of free flaps anastomosed with an AV loop for thoracic wall reconstruction (22 ± 7 s) was significantly lower than that of lower extremity reconstruction cases (52 ± 26 s). Our results suggest that AV loop cases requiring lower extremity reconstruction are at higher risk of hemodynamic complications than that of thoracic wall reconstruction. A closer look at the two cases with postoperative hemodynamic complications showed that their average ITT (46 s) was higher than the average ITT of free flaps without complications (36 s). However, the small number of postoperative complications in this study does not allow for statistical analysis. In general, the combination of AV loop construction and free flap reconstruction is reserved for a highly selective group of patients for whom no other reconstructive option exists. Due to the generally small number of such procedures, knowledge of their hemodynamic properties in the literature is relatively limited. We not only found relevant hemodynamic differences of AV loops and free flaps, depending on the region of reconstruction, but also that the blood flow behavior of myocutaneous flaps remains relatively unaffected by the anastomosis with high flow volume AV loops. This study further enables the determination of standard values for AV loops followed by free flap transfer. The resulting advanced understanding of these hemodynamic properties should further facilitate the intraoperative assessment of vessel patency and flap perfusion.

### Limitations

As this is the first study to investigate hemodynamic parameters of free flaps with high-flow arteriovenous loops, no reference data of such measurements are available in the literature. However, previous studies have performed measurements of several hemodynamic parameters of various free flap types anastomosed to regular anatomically present low-flow recipient vessels, which were compared with our results. The choice of free flap type and AV loop localization depended on wound localization and characteristics, which made a certain selection bias unavoidable.

## 5. Conclusions

In this study, we performed intraoperative measurements in order to determine blood flow and perfusion characteristics of latissimus dorsi and VRAM free flaps anastomosed with AV loops. For this purpose, we have used the combination of Transit-Time Flowmetry with microvascular Indocyanine Green Angiography. This comparative study provides for the first time an evaluation on intraoperative hemodynamic properties of AV loops and free flaps at two different regions of reconstruction. This study serves as groundwork for establishing predictive values for postoperative thrombotic events for this highly selective patient group in the future. In addition, this work may be fundamental for increasing the safety of this particular surgical procedure.

## Figures and Tables

**Table 1:** Measurement time points. AV1: At the apex of the AV loop after construction; F: the isolated flap artery and vein after flap elevation and prior to transfer; AV2: at the apex of the AV loop before its dissection and prior to flap anastomosis; AA: the flap artery and vein after anastomosis with the AV loop. Legend: TTFM = Transit-Time Flowmetry; aVR = arterial Vascular Resistance; mICG-A = microvascular Indocyanine Green Angiography.

Stage	Measurement	TTFM	aVR	mICG-A
1. AV Loop Construction	AV1	x	x	
2. Free Flap Transfer	F	x	x	
	AV2	x	x	
	AA	x	x	x

**Table 2:** Blood flow volume (mL/min) of the flap pedicle in situ (F), the AV loop before flap transfer (AV2) and after anastomosis with the AV loop (AA).

		Flow in mL/in (Mean ± SD)					ITT in Seconds (Mean ± SD)		
		Flap Pedicle in Situ (F)		AV Loop before Flap Transfer (AV2)	After Anastomosis (AA)		After Anastomosis (AA)	*p*-Value	
Type of Flap	No. (%)	Artery	Vein		Artery	Vein		Artery F vs. AA	Artery AV vs. AA
All	11	15.8 ± 6.4	10.6 ± 6.6	698 ± 464	18.5 ± 8.3	18.5 ± 9.3	38 ± 25	0.3	<0.0001
Latissimus	7	16.6 ± 6.9	13 ± 7.1	780 ± 495	16.3 ± 7.4	17 ± 10.3	36 ± 23	0.8	<0.01
VRAM	4	14.5 ± 6.2	6.5 ± 2.6	555 ± 430	22.3 ± 9.5	21 ± 8.1	43 ± 30	0.1	<0.01
*p*-value (Latissimus vs. VRAM)		0.6		0.5	0.3		0.8		

**Table 3:** Blood flow volume (mL/min) of the AV loop at construction (AV1) before flap transfer (AV2) and after anastomosis (AA), as well as the Intrinsic Transit Time (ITT) of free flaps for the reconstruction of the lower extremity and the thoracic wall.

	Flow (Mean ± SD)			ITT (Mean ± SD)	*p*-Value	
AV Loop Site	mL/min			Seconds		
	AV Loop at Construction (AV1)	AV Loop before Flap Transfer (AV2)	After Anastomosis (AA)	After Anastomosis (AA)	Flow AV1 vs. AV2	Flow AV2 vs. AA
All	466 ± 180	698 ± 464	18.5 ± 8.3	38 ± 25	0.1	<0.0001
Lower Extremity	478 ± 174	875 ± 513	22 ± 9.9	52 ± 26	0.2	0.001
Thoracic Wall	453 ± 206	486 ± 324	14.2 ± 2.7	22 ± 7	0.8	0.0002
*p*-value (Lower Extremity vs. Thoracic Wall)	0.6	0.3	0.1	0.03		

## Data Availability

The datasets generated during the current study are available from the corresponding author on reasonable request.

## Supplementary Materials

The following supporting information can be downloaded at: https://www.mdpi.com/article/10.3390/jcm12237477/s1. Table S1: Strobe statement. Table S2: Patient characteristics.
