# Supplementary material for: Intraoperative Blood Flow Analysis of Free Flaps with Arteriovenous Loops for Autologous Microsurgical Reconstruction
Source: J Clin Med. 2023 Dec 2;12(23):7477. doi: 10.3390/jcm12237477 (PMC10707281; doi:10.3390/jcm12237477)
Supplement: Supplementary file 1 [file jcm-12-07477-s001.zip › jcm-2707831-supplementary.pdf]

Supplementary TableS1. STROBE Statement—*Intraoperative Blood Flow Analysis of Free Flaps with Arteriovenous Loops for Autologous Microsurgical Reconstruction* by Geierlehner, A. et al.

	Item No.	Recommendation	Page No.
**Title and abstract**	1	(*a*) Indicate the study's design with a commonly used term in the title or the abstract.	1
		(*b*) Provide in the abstract an informative and balanced summary of what was done and what was found.	1
**Introduction**			
Background/rationale	2	Explain the scientific background and rationale for the investigation being reported.	1, 2
Objectives	3	State specific objectives, including any prespecified hypotheses.	2
**Methods**			
Study design	4	Present key elements of study design early in the paper.	2
Setting	5	Describe the setting, locations, and relevant dates, including periods of recruitment, exposure, follow-up, and data collection.	2, 3
Participants	6	(*a*) *Cohort study*—Give the eligibility criteria, and the sources and methods of selection of participants. Describe methods of follow-up.<br>*Case-control study*—Give the eligibility criteria, and the sources and methods of case ascertainment and control selection. Give the rationale for the choice of cases and controls.<br>*Cross-sectional study*—Give the eligibility criteria, and the sources and methods of selection of participants.	2, 3, 5
		(*b*) *Cohort study*—For matched studies, give matching criteria and number of exposed and unexposed.<br>*Case-control study*—For matched studies, give matching criteria and the number of controls per case.	N/A
Variables	7	Clearly define all outcomes, exposures, predictors, potential confounders, and effect modifiers. Give diagnostic criteria, if applicable.	
Data sources/ measurement	8*	For each variable of interest, give sources of data and details of methods of assessment (measurement). Describe comparability of assessment methods if there is more than one group.	3, 4, 5
Bias	9	Describe any efforts to address potential sources of bias.	3, 4, 5
Study size	10	Explain how the study size was arrived at.	2, 3

Quantitative variables	11	Explain how quantitative variables were handled in the analyses. If applicable, describe which groupings were chosen and why.	6
Statistical methods	12	(*a*) Describe all statistical methods, including those used to control for confounding.	6
		(*b*) Describe any methods used to examine subgroups and interactions.	6
		(*c*) Explain how missing data were addressed.	N/A
		(*d*) *Cohort study*—If applicable, explain how loss to follow-up was addressed.<br>*Case-control study*—If applicable, explain how matching of cases and controls was addressed.<br>*Cross-sectional study*—If applicable, describe analytical methods taking account of sampling strategy.	N/A
		(*e*) Describe any sensitivity analyses.	
**Results**			
Participants	13*	(a) Report numbers of individuals at each stage of study—e.g., numbers potentially eligible, examined for eligibility, confirmed eligible, included in the study, completing follow-up, and analysed.	6, 7, 12, 13
		(b) Give reasons for non-participation at each stage.	N/A
		(c) Consider use of a flow diagram.	N/A
Descriptive data	14*	(a) Give characteristics of study participants (e.g., demographic, clinical, social) and information on exposures and potential confounders.	6
		(b) Indicate number of participants with missing data for each variable of interest.	6
		(c) *Cohort study*—Summarise follow-up time (e.g., average and total amount).	13
Outcome data	15*	*Cohort study*—Report numbers of outcome events or summary measures over time.	6-13
		Case-control study—Report numbers in each exposure category, or summary measures of exposure.	N/A
		Cross-sectional study—Report numbers of outcome events or summary measures.	N/A
Main results	16	(*a*) Give unadjusted estimates and, if applicable, confounder-adjusted estimates and their precision (e.g., 95% confidence interval). Make clear which confounders were adjusted for and why they were included.	6-13
		(*b*) Report category boundaries when continuous variables were categorized.	N/A
		(*c*) If relevant, consider translating estimates of relative risk into absolute risk for a meaningful time period.	N/A

Continued on next page

Other analyses	17	Report other analyses done—e.g., analyses of subgroups and interactions, and sensitivity analyses.	6-13
**Discussion**			
Key results	18	Summarise key results with reference to study objectives.	14-15
Limitations	19	Discuss limitations of the study, taking into account sources of potential bias or imprecision. Discuss both direction and magnitude of any potential bias.	15
Interpretation	20	Give a cautious overall interpretation of results considering objectives, limitations, multiplicity of analyses, results from similar studies, and other relevant evidence.	15
Generalisability	21	Discuss the generalisability (external validity) of the study results.	15
**Other information**			
Funding	22	Give the source of funding and the role of the funders for the present study and, if applicable, for the original study on which the present article is based.	15

*Give information separately for cases and controls in case–control studies and, if applicable, for exposed and unexposed groups in cohort and cross-sectional studies.

Note: An explanation and elaboration article discusses each checklist item and gives methodological background and published examples of transparent reporting. The STROBE checklist is best used in conjunction with this article (freely available on the Web sites of PLoS Medicine at http://www.plosmedicine.org/, Annals of Internal Medicine at http://www.annals.org/, and Epidemiology at http://www.epidem.com/). Information on the STROBE Initiative is available at www.strobe-statement.org.

Supplementary Table S2. Patient characteristics

Patient No.	Age (years)	Defect Location	Type of Flap	AV Loop Site (Vessels)	AV Loop Graft	Time Period (AV Loop Construction - Free Flap Transfer) (Two-staged Procedure; in days)	Flap Weight (grams)	Ischemia Time (minutes)	Intrinsic Transit Time (seconds)
1	65	Lower Extremity	Latissimus dorsi	Femoral	Great Saphenous Vein	6	-	44	50
2	27	Lower Extremity	VRAM	Femoral	Great Saphenous Vein	7	703	83	41
3	66	Thoracic wall	VRAM	Subclavia	Great Saphenous Vein	9	389	58	22
4	74	Thoracic wall	Latissimus dorsi	Subclavia	Great Saphenous Vein	8	361	-	26
5	72	Lower Extremity	Latissimus dorsi	Femoral	Great Saphenous Vein	7	822	43	80
6	36	Lower Extremity	Latissimus dorsi	Femoral	Great Saphenous Vein	7	441	71	33
7	80	Lower Extremity	VRAM	Femoral	Great Saphenous Vein	6	587	65	86
8	80	Lower Extremity	VRAM	Femoral	Great Saphenous Vein	6	924	68	21
9	80	Thoracic wall	Latissimus dorsi	Subclavia	Great Saphenous Vein	7	379	66	16
10	64	Thoracic wall	Latissimus dorsi	Subclavia	Great Saphenous Vein	6	293	54	13
11	56	Thoracic wall	Latissimus dorsi	Subclavia	Great Saphenous Vein	8	347	61	31
